# Toward Colorectal Cancer Biomarkers: The Role of Genetic Variation, Wnt Pathway, and Long Noncoding RNAs

**DOI:** 10.1089/omi.2020.0231

**Published:** 2021-05-07

**Authors:** Amany I. Abdel-Motaleb, Hassan M. Azzazy, Ahmed Moustafa

**Affiliations:** ^1^ Biotechnology Graduate Program, American University in Cairo, New Cairo, Egypt.; ^2^ Department of Chemistry and American University in Cairo, New Cairo, Egypt.; ^3^ Department of Biology, American University in Cairo, New Cairo, Egypt.

**Keywords:** colorectal cancer, Wnt pathway, biomarkers, cancer research, long noncoding RNAs, human genetic variation

## Abstract

Colorectal cancer (CRC) is the third leading cause of death worldwide, comprising nearly 8% of cancer-related deaths per year. In South Korea, for example, CRC is the second most common cancer in men, and third in women. This study reports on the association of CRC with genetic variations in long noncoding RNAs, activators, and inhibitors of a cell proliferation pathway. Five normal colon mucosa tissue samples and their matched five-stage IV CRC samples were evaluated (dataset Gene Expression Omnibus accession: GSE50760). We identified more than 5000 differentially expressed genes (DEGs). The Wnt pathway had the greatest portion of DEGs, including activators, inhibitors, and associated long noncoding RNAs (lncRNAs), suggesting the importance of Wnt pathway in CRC. The following genes were aberrantly expressed: *WIF1*, *SFRP4*, *CD82*, *WNT2*, *WNT3*, *WNT5A*, *HOTAIR*, *CRNDE*, and *UCA1*. Notably, *HOTAIR* is known to silence *WIF1*, and *WIF1* inhibits the Wnt ligands to negatively regulate the pathway. The lncRNA *CRNDE* positively regulates *WNT5A*, while *UCA1* positively regulates *WNT2* and *WNT3*. We note that HOTAIR was unable to silence *WIF1*. *CRNDE* and *UCA1* were found to be upregulated, which may explain the high expression of the *WIF1* targets. Furthermore, 10 single-nucleotide polymorphisms (SNPs) were identified in five of the candidate genes above. A possible novel SNP in *CD82*, chr11:44619242T > C, was predicted to introduce a *ZBTB7A* binding site. These SNPs are hypothesized to contribute to aberrant and discrepant regulation of the Wnt pathway in a context of CRC pathogenesis. These findings collectively inform future research on diagnostics and therapeutics innovation in CRC.

## Introduction

There is a high lifetime risk of developing colorectal cancer (CRC): 4.7% for men and 4.4% for women (Kweon, 2018). The survival rate in patients with localized CRC is over 90%, but drops significantly below 14% when cancer has metastasized (Kweon, 2018). There is an unmet need for diagnostics and therapeutics innovation in the field of oncology and CRC in particular. This study reports on the association of CRC with genetic variations in long noncoding RNAs as well as of activators and inhibitors of a cell proliferation pathway.

### Background integrative biology context for the study

Long noncoding RNAs (lncRNAs) are >200 bp, polyadenylated, and lack an open reading frame. lncRNAs have been reported to have major impact on regulation, epigenetic modifications, and splicing, among other effects in cell biology (Guerrieri, 2015; Li et al., 2017). It is known that the nonprotein coding region of DNA totals 70–90% of the genome; however, very little is known concerning this major component of the eukaryotic genome.

Some notable lncRNAs are HOX transcript antisense RNA (*HOTAIR*), urothelial carcinoma associated 1 (*UCA1*), and colorectal neoplasia differentially expressed (*CRNDE*). *HOTAIR* is one of the first known lncRNAs, which is located on chromosome 12 and comprises nearly 2 kb and six exons (Cai et al., 2014; Hajjari and Salavaty, 2015). It arises from the antisense strand of the *HOXC* gene and is a trans-acting lncRNA that has multiple targets. Its main regulatory role is the alteration of chromatin by recruiting the polycomb repressive complex 2 (*PRC2*) to trimethylate histone H3 on lysine 27 (H3K27) and recruiting lysine-specific demethylase 1 (*LSD1*) to demethylate lysines 4 and 9 on histone H3.

*UCA1* lncRNA is located on chromosome 19 and is 1.4 kb in length (Qian et al., 2017). It was first discovered in bladder cancer. It was found to be upregulated with tumorigenesis and invasion. In addition, it has been documented as a tumor promoter in breast, colorectal, gastric, and esophageal squamous cell carcinoma (Wang et al., 2017). Recently, the upregulation of *UCA1* has been associated with the uncontrolled growth through the p53/p21 signaling pathway (Wang et al., 2017). In oral squamous cell carcinoma, it was found that *UCA1* may also have a relationship with the Wnt signaling pathways.

*CRNDE* is an lncRNA known to be differentially expressed in CRC (Ding et al., 2017; Han et al., 2017). It is located on chromosome 16 on the antisense strand of the gene *IRX5*, contains 10 kb, five exons, and has been shown to be alternatively spliced. *CRNDE* is also found to be overexpressed in various cancers, including, ovarian, pancreatic, liver, kidney, and leukemia. *CRNDE* has been shown to regulate neuronal differentiation, gametogenesis, and cancer progression.

Importantly, the Wnt pathway has been known to have an aberrant expression in all stages of CRC. The key activators of the Wnt pathway are WNT ligands (Abdelmaksoud-Dammak et al., 2014; Huang et al., 2014; Roperch et al., 2013). The WNT ligands bind to the Frizzled (Fzd)/low density lipoprotein receptor (LRP) to signal either the canonical or noncanonical pathways, which in turn will promote cell proliferation, cell adhesion, or cytoskeletal rearrangement.

Inhibitors of the Wnt pathway include Wnt Inhibitory Factor 1 (*WIF1*). *WIF1* is a Wnt antagonist and acts by binding to WNT ligands to prevent binding to the Fzd/LRP receptor complex (Abdelmaksoud-Dammak et al., 2014; Huang et al., 2014; Roperch et al., 2013). Other inhibitors include secreted frizzled-related protein 4 (*SFRP4*), which is a member of the SFRPs and is an inhibitor of the Wnt pathway by targeting the WNT ligands. SFRPs contain a cysteine-rich binding domain, which gives SFRPs the capability to bind to Fzds. This results in a competitive binding between SFRPs and WNT ligands to bind to Fzd. In addition, SFRPs can bind directly with WNT ligands to inhibit their binding to Fzd, thus interfering with the Wnt pathway in two ways.

There are various mutations that account for the upregulation of the Wnt pathway in CRC (Ding et al., 2017; Fearon and Wicha, 2014; Ghorbanoghli et al., 2016; Tanaka et al., 2017; Zhan et al., 2017). Two of the most common mutations occur in the adenomatous polyposis coli (*APC*) gene and *β-catenin*. Mutations in *APC* cause a loss of function. *APC* is a key component in the destruction complex, which targets *β-catenin*. Mutations in *APC* can arise sporadically or originate from a hereditary illness, familial adenomatous polyposis (FAP). Mutations in β-catenin cause a loss of a phosphorylation site in the N-terminus, which is pivotal for the destruction complex to recognize, phosphorylate, and degrade β-catenin. The loss of this site, in turn, allows for constitutive signaling of the canonical Wnt pathway.

Other mutations that are common in CRC consist of mutations in other components of the destruction complex, including *Axin 1* and *2* (Mazzoni et al., 2015; Mazzoni and Fearon, 2014; Novellasdemunt et al., 2015). *Axin1* has a ubiquitous expression across all tissue types, while *Axin2* is developmentally and tissue specific; furthermore, *Axin2* is a target of the Wnt pathway which creates a negative feedback loop.

Related to the Wnt Pathway, *CD82* belongs to the tetraspanin family of proteins and is a tumor metastasis suppressor that has been found to be inversely correlated with cell invasion, motility, metastasis, and cell survival (Aram et al., 2017; Challa et al., 2014; Wu et al., 2014; Zhu et al., 2017). *CD82* was first discovered in 1995 as a tumor metastasis suppressor in rats for the treatment of prostate cancer (Dong et al., 1995). Since then, recent studies have reported metastasis suppression capabilities in bladder, breast, hepatocellular, and pancreatic cancers (Zhu et al., 2017). *CD82* acts in accordance with integrins and receptor tyrosine kinases; however, its exact mechanism is unclear.

Stage IV CRC exhibits the classic metastasized attributes of invasion, uncontrolled cell proliferation, deletions, mutations, and dysregulation of various pathways. Further investigation is needed to describe the underlying mechanisms that regulate lncRNAs and the role of lncRNAs in oncogenesis to fully understand the scope and environment of metastasized CRCs.

## Materials and Methods

### Data source and sample selection

Data analyzed in this study were obtained from the Gene Expression Omnibus (GEO; with the accession number GSE50760), initially published by Kim et al., in 2014. The original study used matched tissue samples from 18 patients with primary stage IV CRC cancer and liver metastasis. Three tissue samples from each patient were sequenced: primary CRC tissue, metastatic liver tissue, and normal colon tissue. All patients were treated in the Asan Medical Center in Seoul, South Korea, between May 2011 and February 2012 (Kim et al., 2014).

### RNA-seq data processing

In this study, five paired primary cancer tissue datasets and five normal tissue datasets were studied. The reads were paired-end reads, which generated two reads per sample, resulting in 10 normal tissue replicates and 10 cancer tissue replicates. These sequence reads were processed by splitting the ends and trimming and filtering poor-quality reads. The processed reads were then assessed for quality using FastQC v 0.11.2 (www.bioinformatics.babraham.ac.uk/projects/fastqc/).

### RNA-seq alignment and differential expression

Following the initial processing, the Tuxedo Suite Package was used to determine the differentially expressed genes (DEGs) from the processed RNA sequences, from paired normal and cancerous tissues. First, the processed reads were aligned to the human genome Hg38 using Bowtie v.2.2.6.0 (www.bowtie-bio.sourceforge.net/index.shtml) and TopHat v.2.1.0 (https://www.ccb.jhu.edu/software/tophat/index.html) (Trapnell et al., 2009). Then, the sequences were assembled into transcripts using Cufflinks v2.2.1 (www.cole-trapnell-lab.github.io/cufflinks/install); the transcripts for all replicates are merged by CuffMerge v2.0.2 (www.cole-trapnell-lab.github.io/cufflinks/cuffmerge/) (Roberts et al., 2011; Trapnell et al., 2017). The differentially expressed transcripts were determined using CuffDiff v2.2.2 (www.cole-trapnell-lab.github.io/cufflinks/cuffdiff/), and finally, the differential expression patterns were visualized using an R package, CummeRbund v3.2.3 (www.compbio.mit.edu/cummeRbund/) (Thomas et al., 2003; Trapnell et al., 2012a). Statistical analysis of the DEGs was visualized with scatterplots and volcano plots shown in Supplementary Figures S2 and S3.

### Differential gene analysis

To classify the DEGs, the PANTHER database (v 11.1) was used. PANTHER is available online at www.pantherdb.org/about.jsp (Thomas et al., 2003). PANTHER is an acronym for Protein ANalysis THrough Evolutionary Relationships and is a classification tool designed to analyze high-throughput datasets. PANTHER is part of the Gene Ontology Phylogenetic Annotation Project and contains 15,702 protein families, divided into 123,989 functionally distinct protein subfamilies. The genes are classified in four ways: family and subfamily, molecular function, biochemical process, and pathway. Three sets of genes were analyzed: all DEGs, upregulated DEGs, and downregulated DEGs. A pipeline summarizing the analysis is shown in Supplementary Figure S4.

lncRNA2Target (v 1.2) is a web tool that finds the lncRNA that targets the gene of interest or finds what genes particular lncRNA targets (Jiang et al., 2015). Focusing on the WNT proteins and WNT inhibitors, a table of associated lncRNAs was established (Tables 1 and 2).

**Table 1.** lncRNA2Target Results for Differentially Expressed Wnt Ligands (Jiang et al., 2015)

WIF1 target	Fold change	*p*-Value	Associated lncRNA	Fold change	*p*-Value	lncRNA action
*Wnt9A*	−1.50	0.00075	None	—	—	—
*Wnt3*	1.50	5.00E-05	*CRNDE*	3.22	0.0032	Positively regulates
*Wnt11*	1.99	5.00E-05	*TUG1*	—	—	Positively regulates
*Wnt5A*	2.26	5.00E-05	*UCA1*	5.54	5.00E-05	Positively regulates
*Wnt2*	5.72	0.0019	*CRNDE*	3.22	0.0032	Positively regulates

The differentially expressed Wnt ligands were entered into lncRNA2Target database to find associated lncRNAs. *WNT9A* has no known associated lncRNAs. lncRNA *CRNDE* is known to activate *WNT2* and *WNT3*. *TUG1* is known to target *WNT11*; however, *TUG1* is not differentially expressed in this dataset. *UCA1* is known to target and activate *WNT5A*.

CRNDE, colorectal neoplasia differentially expressed; lncRNA, long noncoding RNA; UCA1, urothelial carcinoma associated 1; WIF1, Wnt inhibitory factor 1.

**Table 2.** The Identification of Differentially Expressed Long Noncoding RNAs Associated with Wnt Inhibitors

Wnt inhibitors	Fold change	*p*-Value	Associated lncRNA	Associated lncRNA	Fold change	*p*-Value
*SFRP4*	2.12	0.00185	*CRNDE*	Negatively regulates	3.22	0.0032
*APCDD1*	2.38	5.00E-05	*MALAT1*	Negatively regulates	—	—
*DKK2*	4.34	5.00E-05	*TUG1*	Positively regulates	—	—
*WIF1*	5.16	5.00E-05	*HOTAIR*		5.19	0.03785
*NOTUM*	6.81	0.00115	None		—	—
*DKK4*	INF	0.00175	None		—	—

To find associated lncRNAs, the differentially expressed Wnt inhibitors were entered into the lncRNA2Target database (Jiang et al., 2015). *CRNDE* is known to negatively regulate *SFRP4*. *MALAT1* is known to negatively regulate *APCDD1*; however, *MALAT1* is not differentially expressed in this dataset. *TUG1* is known to positively regulate *DKK2*; however, *TUG1* is not differentially expressed. *HOTAIR* is known to negatively regulate *WIF1*. There are no known lncRNAs associated with *NOTUM* or *DKK4*.

HOTAIR, HOX transcript antisense RNA; SFRP4, secreted frizzled-related protein 4.

The Integrative Genomics Viewer (IGV) (v 2.3.97) was used to visualize transcripts and sequences of the data (Robinson et al., 2011).

Once a single-nucleotide polymorphism (SNP) was found in IGV, Single Nucleotide Polymorphism Database (dbSNP) was used to determine the novelty or if the SNP was previously recorded (Sherry et al., 2001).

The SNPs are then further evaluated for function and consequence with FuncPred (Xu and Taylor, 2009).

To find the SNP frequencies and determine whether these SNPs are unique to the South Korean and Asian populations, dbSNP was also used to compare SNP frequencies with the global average.

## Results

### Differential gene expression and pathway enrichment analysis

We identified more than 5000 DEGs. Pathway enrichment was done thrice with the complete set of DEGs/ORFs (collectively abbreviated as DEGs), the set of upregulated genes, and the set of downregulated genes using the PANTHER Database.

**FIG. 1.**
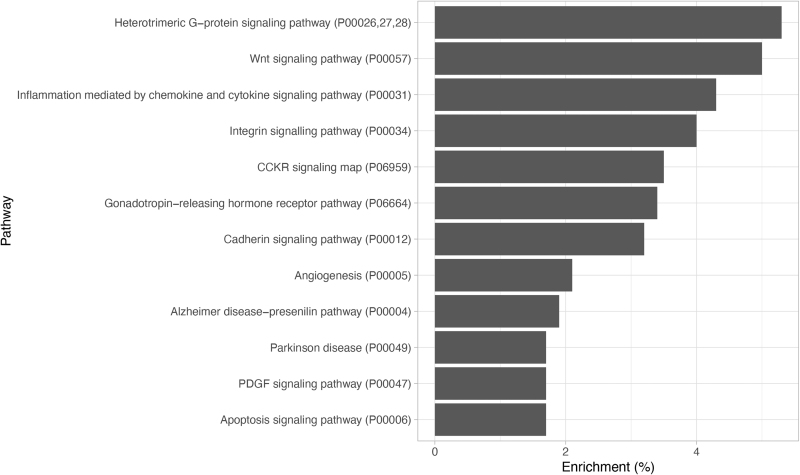
Pathway enrichment analysis in DEGs in colorectal cancer samples. The top 12 enriched pathways are listed with their PANTHER accessions (Thomas et al., 2003). The most enriched pathway was the heterotrimeric G-protein signaling pathway comprising 5.1%, followed by the Wnt pathway comprising 5% of all the DEGs. DEG, differentially expressed gene.

Figure 1 shows the top 10 most enriched pathways in this dataset. The top three pathways were the heterotrimeric G-protein signaling pathway, Wnt pathway, and inflammation mediated by chemokine and cytokine signaling pathway. Shown in Supplementary Figure S1 are the results of the upregulated and downregulated genes. Of the upregulated genes, the Wnt pathway genes represented the most enriched set of genes with 7.2%. Of the downregulated genes, the Wnt pathway genes represented the fifth most enriched set of genes only representing 2.5% of the genes.

The Wnt pathway is active in the cancer dataset according to the number and the fold-change values of Wnt ligands and the Wnt pathway targets. Of the various known Wnt ligands, five were differentially expressed: *WNT9A*, *WNT3*, *WNT11*, *WNT5A*, and *WNT2* with fold-change values of −1.503, 1.503, 1.996, 2.261, and 5.722, respectively (Supplementary Fig. S5). Interestingly, it was found that there were five inhibitors that also had high expression: *SFRP4*, *APCDD1*, *DKK2*, *WIF1*, and *NOTUM* with fold-change values of 2.211, 2.384, 4.342, 5.165, and 6.812, respectively (Supplementary Fig. S5).

### lncRNA target analysis

Six inhibitors, five WNT ligands, and their associated lncRNAs were found through lncRNA2Target database. The results are shown in Tables 1 and 2, respectively. *WIF1* (fold-change value: 5.165) is targeted by *HOTAIR* (fold-change value: 5.198) for methylation. *SFRP4* (fold-change value: 2.121) is targeted by *CRNDE* (fold-change value: 3.227) for negative regulation by an unknown mechanism. *WNT2* (fold-change value: 5.722) and *WNT3* (fold-change value: 1.503) are targeted by CRNDE for positive regulation by an unknown mechanism. *WNT5A* (fold-change value: 2.261) is targeted by *UCA1* (fold-change value: 5.542) for positive regulation by an unknown mechanism.

Since the mechanism of *HOTAIR* is well studied and had a high fold-change value, the targets of *HOTAIR* were found with the PANTHER database (Cantile et al., 2021; Chao et al., 2020). Shown in Table 3, some known targets of *HOTAIR* were differentially expressed. Out of the six differentially expressed targets, four showed the expected expression, while two showed the opposite of the expected (Table 3). The two targets, *WIF1* and *CD82* (fold-change value: 5.16 and 1.05, respectively), showed the opposite of the expected expression pattern. Under normal conditions, *WIF1* and *CD82* are expected to be silenced by *HOTAIR*; in this dataset, *WIF1* and *CD82* are upregulated (Jiang et al., 2015).

**Table 3.** Differential Expression of *HOTAIR* Target Genes

Gene	Expected effect	Observed effect	Expression value	*p*-Value
*MMP1*	↑	↑	4.70	5.0E-5
*MMP3*	↑	↑	5.54	5.0E-5
*MMP9*	↑	↑	1.42	5.0E-5
*VEGFA*	↑	↑	1.06	0.0004
*WIF1*	***↓***	**↑**	**5.16**	**5.0E-5**
*CD82*	***↓***	**↑**	**1.05**	**0.00075**

A list of *HOTAIR* target genes was obtained from lncRNA2Target database (Jiang et al., 2015). This list was cross referenced with the DEGs (Supplementary Table S1) and a table was generated showing the expected and observed effect with the corresponding expression value and *p*-value. *WIF1* and *CD82* are two targets where the expected and observed effects were not correlated, in which the observed effect was opposite to the expected (bolded).

DEG, differentially expressed gene.

### Genetic variation analysis

Normal and cancer datasets were visually investigated using IGV for the following genes: *WIF1*, *SFRP4*, *WNT2*, *WNT3*, *WNT5A*, *CRNDE*, *UCA1*, *HOTAIR*, and *CD82*. Ten SNPs (Table 4) were found in five of the genes: *WIF1*, *SFRP4*, *WNT5A*, *UCA1*, and *CD82*. No consistent abnormalities were found in the others. Nine of the 10 SNPs were previously recorded in dbSNP, and 1 appears to be novel in *CD82* (bolded in Table 4).

**Table 4.** The Investigation of the Presence of Single-Nucleotide Polymorphisms in the Genes of Interests

Number	Gene	Observed SNP location	Accession no.	Consequence	No. of replicates containing SNP (%)
**1**	*CD82*	**chr11:44,619,242**	**NA**		**100**
2		chr11:44,619,090	rs7107335	Synonymous	40
3		chr11:44,618,718	rs11541053	Missense	100
4	*SFRP4*	chr7:37,907,562	rs1802073	Missense	70
5		chr7:37,906,899	rs1052981	3′UTR	50
6		chr7:37,912,124	rs1132553	Synonymous	80
7		chr7:37,914,238	rs1132552	Synonymous	100
8	*UCA1*	chr19:15,834,304	rs7258210	Intron variant	70
9	*WIF1*	chr12:65,120,486	rs7301320	Synonymous	40
10	*WNT5A*	chr3:55,466,974	rs669889	3′UTR	70

All genes of interest were analyzed in IGV for abnormalities in the transcripts, *CD82*, *SFRP4*, *UCA1*, *WIF1*, and *WNT5A* showed consistent SNPs throughout the samples (Robinson et al. 2011). Nine out of the 10 observed SNPs were previously recorded and found in dbSNP, the bolded SNP *CD82* was novel at location chr11:44,619,242 with a frequency in 100% of the samples.

dbSNP, Single Nucleotide Polymorphism Database; IGV, Integrative Genomics Viewer; NA, not applicable; SNP, single-nucleotide polymorphism.

The SNPs were arbitrarily named for function and simplicity. *WIF1* had a synonymous SNP with the accession number rs7301320 (WIF1-S1) present in four of the sample datasets. *SFRP4* had four SNPs, rs1802073 (missense) (SFRP4-M1), rs1052981 (3′UTR) (SFRP4-3), rs1132553 (synonymous) (SFRP4-S1), and rs1132552 (synonymous) (SFRP4-S2), which were present in 7, 5, 8, and 10 of the sample datasets, respectively. *WNT5A* had one SNP, rs669889 (3′UTR) (WNT5A-3), present in seven of the sample datasets. *UCA1* had one SNP, rs7258210 (intron variant) (UCA1-IV1), present in seven of the sample datasets. *CD82* had three SNPs, two previously identified rs7107335 (synonymous) (CD82-S1) and rs11541053 (missense) (CD82-M1), which were present in 4 and 10 of the sample datasets, respectively. A possible novel SNP (CD82-N) was also identified in *CD82* at the location chr11:44,619,242 and was present in 10 of the sample datasets.

Using FuncPred prediction software, any possible adverse effect of the SNPs and the possible affected binding sites was predicted (Table 5). For the two known SNPs in *CD82*, CD82-S1 and CD82-S2, both were found to be present in a splice site; however, CD82-M1 was found to be benign. The SNP in *UCA1*, UCA1-IV, was found to be in a transcription factor binding site. SFRP4-3 was found to be in a miRNA binding site. SFRP4-S1 and SFRP4-S2 were found to be in a splicing site. SFRP4-M1 was found to be possibly damaging. WNT5A-3 was found to be in a miRNA binding site. WIF1-S1 was not found to have any adverse effect.

**Table 5.** Predicted Effects of Previously Known Observed Single-Nucleotide Polymorphisms

SNP	rs	TFBS	Splicing	miRNA	nsSNP	Stop codon	Polyphen	Reg potential	Conservation
CD82-M1	rs1139971	—	Y	—	Y	—	Benign	0.49	0.99
CD82-S1	rs7107335	—	Y	—	—	—	—	0.45	0.94
UCA1-IV	rs7258210	Y	—	—	—	—	—	NA	0
SFRP4-3	rs1052981	—	—	Y	—	—	—	0.03	0.002
SFRP4-S2	rs1132552	—	Y	—	—	—	—	0.22	0.80
SFRP4-S1	rs1132553	—	Y	—	—	—	—	0.35	1
SFRP4-M1	rs1802073	—	—	—	Y	—	Possibly damaging	0.41	0.03
WIF1-S1	rs7301320	—	—	—	—	—	—	0.29	0.98
WNT5A-3	rs669889	—	—	Y	—	—	—	0.12	0

Any possible downsteam effects were predicted using FuncPred program (https://www.funcpred.com/). The nine known SNPs were fed into the FuncPred for prediction of potential effects. The SNP in the *UCA1* gene affects a TFBS, two SNPs in *CD82* and two in *SFRP4* affect a splicing mechanism, one SNP in *SFRP4* and one in *WNT5A* are in a miRNA binding region, one SNP in *SFRP4* shows that it is possibly damaging, but there is not a known reason for this hypothesis, and the SNP in *WIF1* shows no adverse consequence.

Y: Yes; —: No; PolyPhen: polymorphism phenotype; Reg Potential: likelihood of transcription factor recognition/regulation; Conservation: score based on conservation of gene throughout 17 species.

nsSNP, non-synonymous SNP; TFBS, transcription factor binding site.

**FIG. 2.**
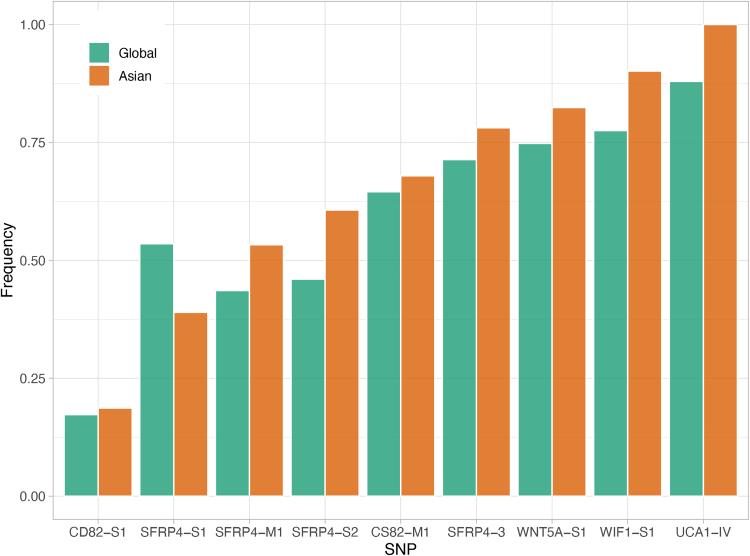
Global and Asian SNP frequency comparison. By using CuffDiff to compare the SNPs found in the colorectal cancer samples with the global population, it was found that the Asian population frequencies are either similar to or higher than the global average, except for SFRP4-S1 (Trapnell et al., 2012b). This SNP has a lower frequency in Asian populations with a score of 0.3898, compared to the global average score of 0.53518. SNP, single-nucleotide polymorphism.

By investigating the SNP frequencies in the Asian population and comparing it with the global average, we hypothesized which SNP may play a role in cancer progression in stage IV CRC (Fig. 2).

## Discussion

There are intensive efforts to identify new molecular targets for CRC with an eye to diagnostics and therapeutics innovation. In the original sample set published in 2014 by Kim et al. using RNA-seq methods, they found 19 genes associated with CRC aggressiveness and developed a risk-score classifier to predict response to adjuvant chemotherapy in four independent cohorts. The present study offers several important leads in this context.

The Wnt pathway can be divided into three subtypes: Wnt/β-catenin or canonical pathway, noncanonical planar cell polarity pathway, and noncanonical Wnt/Calcium pathways (Tabatabai et al., 2017). These pathways promote cell proliferation, cell polarity, and cell adhesion and migration, respectively. The Wnt pathway is known to be upregulated in several types of cancers, including, colorectal, breast, liver, and gastric, among others.

We identified 5303 DEGs/ORFs. Of the 5303 DEGs, 3070 genes/ORFs were upregulated and 2233 were downregulated. Pathway enrichment was performed three times on DEGs, upregulated and downregulated genes/ORFs. Figure 1 shows the top 10 enriched pathways for all the DEGs. Shown in Supplementary Figure S1A are the pathway enrichment results for the upregulated DEGs, which show that the Wnt pathway genes make for the second greatest portion of the total pathways expressed after the heterotrimeric G-protein signaling pathway. Supplementary Figure S1B shows the downregulated DEG pathway enrichment results, in which the Wnt pathway genes were ranked number five; the pathway with the greatest number of genes expressed is cytokine- and chemokine-mediated inflammation. By using this analysis, it is clear that the Wnt pathway plays a major role in stage IV CRC.

The heterotrimeric G-protein signaling pathway has been shown to be an activator of both the canonical and noncanonical Wnt pathways (Shevtsov et al., 2006). In addition, the *FZD* receptor is considered a G protein-coupled receptor and other G proteins modulate *FZD* activity. In the canonical pathway, upon *FZD* activation by a hypertrophic agonist, G proteins G_q_ and G_i_ stabilize *β-catenin* in the nucleus (Avasarala et al., 2013; Wang and Malbon, 2004). When prostaglandin E2 activates *FZD*, the α subunit of the G_s_ protein binds to *Axin*, which leads to the stabilization of β-catenin in the nucleus. In the noncanonical pathways, it has been shown that the G_aq_ family can induce and modulate the mitogen-activated protein kinase (MAPK) pathway (Avasarala et al., 2013).

Shown in the downregulated genes pathway enrichment, there is an inverse relationship between the cytokine- and chemokine-mediated the inflammation pathway and the Wnt pathway (Fig. 1). This inverse relationship between these pathways demonstrates a crosstalk between these two pathways, which has been reported previously in CRC cell lines (Ma and Hottiger, 2016). It appears that the upregulation of the Wnt pathway genes has a negative effect on the cytokine- and chemokine-mediated inflammation or the nuclear factor kappa B (NFκB) pathway (Ma and Hottiger, 2016). It has been shown that the relationship between the Wnt pathway and the NFκB pathway is highly dependent on the cellular context. In this analysis, it appears that the Wnt pathway negatively regulates the NFκB pathway. This is shown by the pathway enrichment results in the Supplementary Figure S1. In this dataset, this pathway represents the most genes in the downregulated gene set. It has been shown that the Wnt pathway can negatively regulate the NFκB pathway through the accumulation of β-catenin. β-Catenin forms a complex with RelA and p50, which is responsible for facilitating DNA binding with NFκB. In turn, NFκB is unable to transcribe its target genes. This relationship is consistent with the previously reported findings in colon cancer cell lines (Du et al., 2009; Liu et al., 2013; Moreau et al., 2011). The crosstalk and regulation of these two pathways have been shown to promote tumorigenesis in several cancers, including CRC (Ma and Hottiger, 2016).

To further investigate the effects of the upregulated Wnt pathway, the ligands/activators and inhibitors were studied along with their associated lncRNAs. The associations between these genes and the lncRNAs were determined by the lncRNA2Target database. lncRNAs associated with the Wnt ligands and Wnt inhibitors and the targets of *HOTAIR* were found and crossed referenced with the DEG list (Supplementary Table S1). All the targets of *HOTAIR* showed the expected expression pattern when *HOTAIR* is expressed, except for *WIF1* and *CD82*. *WIF1* and *CD82* are expected to be downregulated in the presence of *HOTAIR*; however, in this dataset, *WIF1* and *CD82* are upregulated at 5.165 and 1.05, respectively.

*HOTAIR'*s functions are well studied and widely diverse. In short, *HOTAIR* epigenetically silences its targets through the *PRC2* for H3K27 methylation (Cai et al., 2014; Xue et al., 2015). The catalytically active subunit, EZH2, of the *PRC2* is differentially expressed; however, the remaining two subunits, SUZ12 and EED, are not differentially expressed. They are, however, expressed in both normal and cancer conditions.

A recent study showed that *HOTAIR* is able to work independent of *PRC2* in a breast cancer cell line (Portoso et al., 2017). Using forceful overexpression of *HOTAIR*, subtle transcriptomic changes were observed. In addition, they found that by the use of artificial tethering of *HOTAIR* to chromatin, transcriptional repression was observed, but not with the use of *PRC2.*

The target of *HOTAIR* that was aberrantly expressed, *WIF1*, is a proliferation inhibitor and, *CD82* is a metastasis inhibitor (Ramachandran et al., 2014; Zhu et al., 2017). When expressed, the expected phenotypes are of normal tissue homeostatic growth. It was found that these two genes, *WIF1* and *CD82*, carry SNPs. *WIF1* has a single synonymous SNP in 4 of the replicates, while *CD82* has 3 SNPs, 1 novel, 1 synonymous, and 1 missense in 10, 4, and 10 of the replicates, respectively. According to FuncPred, CD82-M1 and CD82-S1 cause a change in the splicing site that causes either a splice site enhancer or silencer region. In addition, CD82-S1 is a benign mutation. Whether *HOTAIR* uses the *PRC2* to silence its targets or not, it would be expected with such a high expression of *HOTAIR* that *WIF1* and *CD82* would be silenced. These SNPs may be the cause or may play a role in its inability to be silenced by *HOTAIR*.

The lncRNA target analysis revealed that lncRNA *CRNDE* negatively affects *SFRP4*. The mechanism by which *CRNDE* accomplishes this is unknown. In this dataset, *CRNDE* and *SFRP4* are upregulated at 3.228 and 2.121, respectively, clearly indicating another factor disrupting this relationship. By IGV analysis, it was found that *SFRP4* carries four SNPs in the cancer sample datasets. SFRP4-S1 and SFRP4-S2 were found to cause changes in splicing site, which may cause a splice site enhancer or silencer, SFRP4-M1 was found to be possibly damaging, but the mechanism of how this could be is not elucidated, and SFRP4-3 was found to have a miRNA binding site. From this analysis, it may be assumed that the SNPs play a role in the dysfunction of *SFRP4*, its regulation by *CRNDE* and miRNAs, and splicing in which contributes to the tumorigenesis of CRC.

**FIG. 3.**
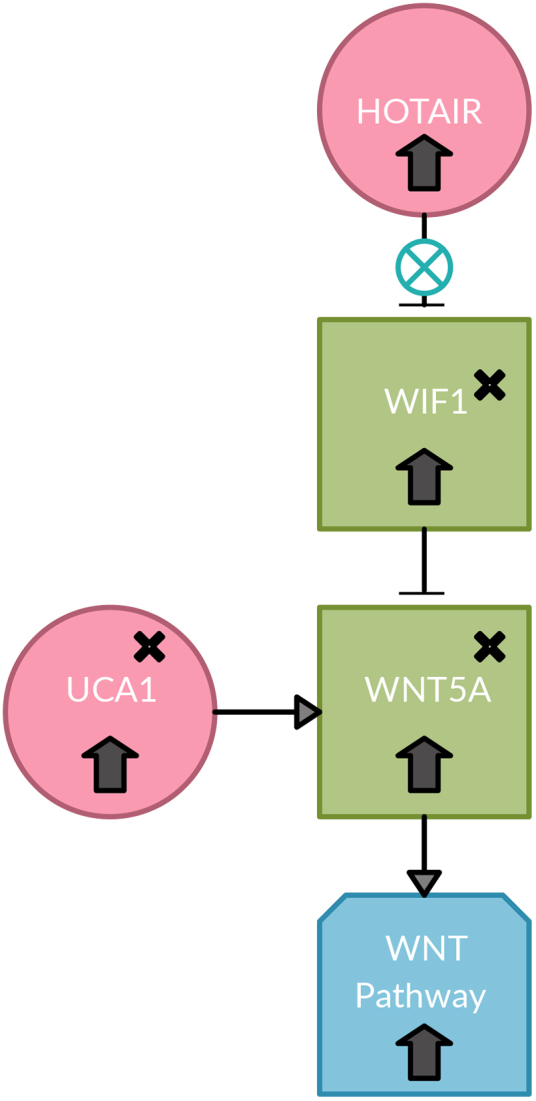
Possible competition mechanism between WIF1 and UCA1 to inhibit or activate WNT5A. With the upregulation of HOTAIR and its inability to silence WIF1, there may be a mutation in the WIF1 promoter that prevents the recognition of WIF1 by HOTAIR, which is observed in this dataset. That would subsequently cause an upregulation of WIF1, which would inhibit its target, WNT5A. WNT5A and its associated lncRNA UCA1 are upregulated. There may be a competition between WIF1 and WNT5A associated with lncRNA, UCA1. UCA1 is known to positively regulate WNT5A. With the upregulation of HOTAIR, WIF1, WNT5A, UCA1, and the Wnt pathway, UCA1 may be competing with WIF1 to positively regulate WNT5A and subsequently the Wnt pathway. The x's indicates this gene contains an SNP found in IGV. WIF1—WIF1-S1 (rs7301320); SFRP4—SFRP4-M1 (rs1802073), SFRP4-3 (rs1052981), SFRP4-S1 (rs1132553), SFRP4-S2 (rs1132552). HOTAIR, HOX transcript antisense RNA; IGV, Integrative Genomics Viewer; lncRNA, long noncoding RNA; UCA1, urothelial carcinoma associated 1; WIF1, Wnt inhibitory factor 1.

Since WIF1-S1 does not cause any structural abnormalities that would inhibit the function of *WIF1* and there are no known post-translational modifications that would degrade *WIF1*, *WIF1* is expected to be active and to inhibit its Wnt ligand targets. Using the lncRNA2Target database, it was found that lncRNA *UCA1* positively regulates *WNT5A* (Table 1). A possible mechanism for cancer to resist or to minimize the effects of the defense mechanism of the cell would be to upregulate a competitor of *WIF1* to ultimately activate the Wnt pathway. The mechanism by which *UCA1* positively regulates *WNT5A* is unknown; however, it is possible that *WIF1* and *UCA1* may compete to inhibit or activate *WNT5A*, respectively (Fig. 3). This mechanism would explain why although *WIF1* is highly expressed, the Wnt pathway is able to transcribe and activate its targets.

**FIG. 4.**
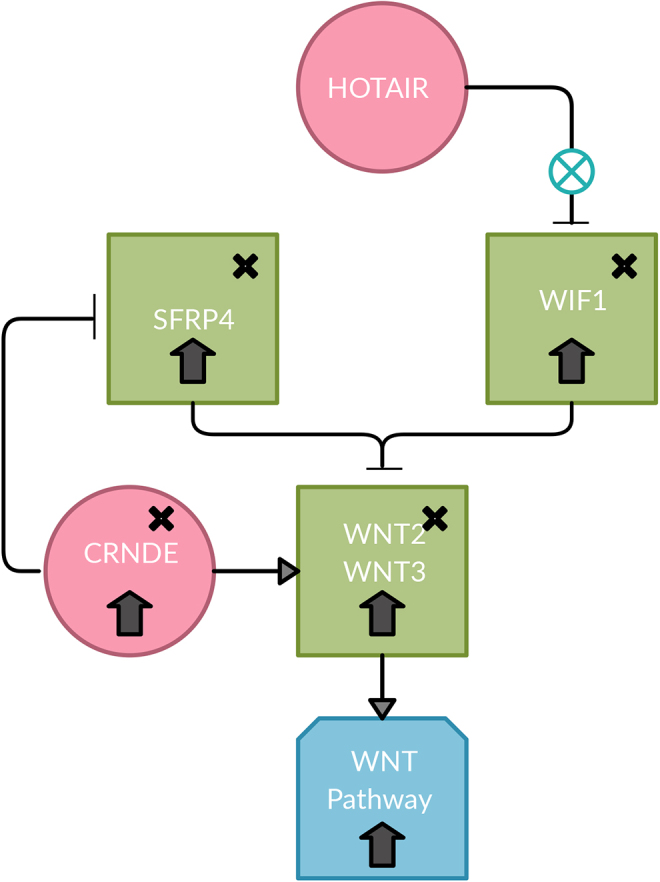
Possible competition mechanism between WIF1 and CRNDE to inhibit or activate WNT2 and WNT3. With the upregulation of HOTAIR and its inability to silence WIF1, there may be a mutation in the WIF1 promoter that prevents the recognition of WIF1 by HOTAIR, which is observed in this dataset. That would subsequently cause an upregulation of WIF1, which would inhibit its targets, WNT2 and WNT3. WNT2 and WNT3 and its associated lncRNA CRNDE are upregulated. There may be a competition between WIF1, CRNDE, and SFRP4. CRNDE is known to positively regulate WNT2 and WNT3 and negatively regulate SFRP4. SFRP4 is known to inhibit WNT2 and WNT3. With the upregulation of HOTAIR, WIF1, WNT2, WNT3, CRNDE, and SFRP4, and the Wnt pathway, CRNDE may be competing with WIF1 to positively regulate WNT2 and WNT3 and the SNP in SFRP4 may be the reason that CRNDE is preventing the inhibition action of SFRP4 and subsequently the Wnt pathway. The x's indicates this gene contains an SNP found in IGV. WIF1—WIF1-S1 (rs7301320); SFRP4—SFRP4-M1 (rs1802073), SFRP4-3 (rs1052981), SFRP4-S1 (rs1132553), SFRP4-S2 (rs1132552). CRNDE, colorectal neoplasia differentially expressed; SFRP4, secreted frizzled-related protein 4.

**FIG. 5.**
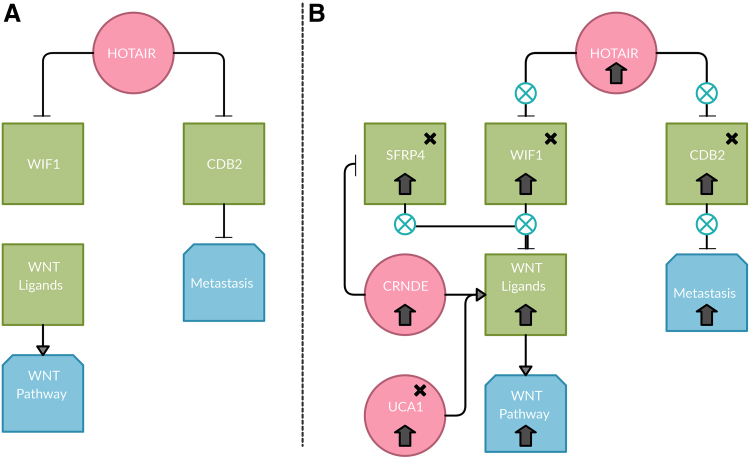
Comparison of pathway outcomes between normal and cancer conditions. On the *left* **(A)** is a schematic of events when HOTAIR is expressed. When HOTAIR is expressed, WIF1 and CD82 are silenced; thus, the Wnt Pathway and metastatic pathways are active. In this case, when HOTAIR is expressed on the *right* **(B)**, the SNPs in WIF1 and CD82 may be preventing HOTAIR from silencing. In normal cases when WIF1 and SFRP4 are expressed, they may bear competing with CRNDE and UCA1 to either inhibit or activate the WNT ligands. What is observed in the samples is that and CRNDE and UCA1 are activating the WNT ligands for the Wnt pathway to be active. In addition, CRNDE may be negatively regulating SFRP4, which may be a contributing factor to the inability of SFRP4 to inhibit the WNT ligands. The x's indicates this gene contains an SNP found in IGV. WIF1—WIF1-S1 (rs7301320); WNT5A—WNT5A-3 (rs669889); UCA1—UCA1-IV (rs7258210); SFRP4—SFRP4-M1 (rs1802073), SFRP4-3 (rs1052981), SFRP4-S1 (rs1132553), SFRP4-S2 (rs1132552); CD82—CD82-S1 (rs7107335), CD82-M1 (rs11541053).

For the *WNT2* and *WNT3* ligands, it was found using lncRNA2Target that lncRNA *CRNDE* positively regulates *WNT2* and *WNT3*; however, the mechanism of which *CRNDE* positively regulates *WNT2* and *WNT3* is unknown. Because the *WIF1* protein would remain unchanged, it is possible that *CRNDE* would also compete with *WIF1* to activate or inhibit *WNT2* and *WNT3* (Fig. 4). This mechanism would explain why the Wnt pathway is upregulated. Figure 5 shows a complete overview of the genes involved with SNPs denoted.

The Asian and global frequencies of the nine known SNPs were found using dbSNP. The two frequencies were compared to determine which SNPs are less prevalent in the Asian population relative to the global average using CuffDiff (Trapnell et al., 2012b). Figure 2 shows the comparison of frequencies for each SNP. Overall, the Asian population frequencies are either similar to or higher than the global average, except for SFRP4-S1. This SNP has a lower frequency in Asian populations with a score of 0.3898, compared to the global average score of 0.53518. *SFRP4* is a WNT ligand inhibitor and the SNP present is synonymous, so the structure is not altered; however, the SNP could alter binding sites or recognition sites. In this case, a site for *CRNDE* to recognize and inhibit *SFRP4* could be created as a result of this SNP. The low frequency shows that there may be a correlation between the SNP and *SFRP4*'s lack of inhibitory function. To verify this information, the mechanism by which *CRNDE* operates needs to be fully elucidated. There is a global change in diet where most populations are switching toward the Western diet and lifestyle, which consists of a high fat, high carbohydrate diet and minimal exercise. The fact that diet and lifestyle are the two biggest factors in CRC explains the similarity in SNP frequencies among Asians and the global average (Khil et al., 2021; Wang et al., 2020).

There are no previous studies, to the best of our knowledge, which show a relationship or connection between WNT ligands, activators, and lncRNAs in stage IV CRC. This study also showed the genetic variants that could be affecting the effectiveness of the genes of interest and how these variants affect the overall pathway. Previous study focused on variants in the *APC*, *Axin 1 and 2*, *FAP*, and *β-catenin* genes (Fearon and Wicha, 2014; Ghorbanoghli et al., 2016; Mazzoni et al., 2015; Mazzoni and Fearon, 2014; Novellasdemunt et al., 2015; Tanaka et al., 2017).

Taken together, this study investigated the relationships between lncRNAs, activators, and inhibitors and how genetic variations in these players may affect cell proliferation pathways. It was found that the Wnt pathway was differentially expressed and within this gene set, several activators (*WNT2*, *WNT3*, and *WNT5A*) and inhibitors (*WIF1* and *SFRP4*) were upregulated. Further investigation showed that there were three lncRNAs associated with these activators and inhibitors, *HOTAIR*, *CRNDE*, and *UCA1*. Differentially expressed *HOTAIR* targets were extracted and examined whether *HOTAIR* effectively asserted its action. These results showed that *WIF1* and *CD82* expressed the opposite of the expected effect of *HOTAIR*. When examining genetic variations, 10 SNPs were found in *WIF1*, *SFRP4*, *WNT5A*, *UCA1*, and *CD82*. Nine of the 10 SNPs found have been previously recorded and 1 appears to be novel in *CD82*.

The aberrant expression of the activators and inhibitors coupled with the accumulation of these SNPs indicated a competition between *WIF1* and *CRNDE* to activate *WNT2* and *WNT3*, and between *WIF1* and *UCA1* to ultimately activate *WNT5A*. Furthermore, *CRNDE* is known to negatively regulate the Wnt inhibitor, *SFRP4*, and in turn, *SFRP4* inhibits the WNT ligands. In this dataset, *SFRP4* has four SNPs and is upregulated along with *CRNDE*. Therefore, the accumulation of the SNPs in *SFRP4* may be responsible for its lack of function in inhibiting the WNT ligands and may be acted upon by *CRNDE*. Finally, the novel SNP may have created a *ZBTB7A* binding site, which may be the one of the causes for the lack of metastasis suppression by *CD82*. The SNPs accumulated in *CD82* have indicated that they may contribute to its lack of metastasis suppressor ability.

Because only transcriptomes from five South Korean males were analyzed in this study, future studies should confirm these findings with all the dataset samples available in the GEO Dataset GSE50760 and compare findings with females and males in other populations. In addition, future studies should confirm the mutation in *WIF1*, *SFRP4*, *WNT5A*, *UCA1*, and *CD82* by DNA sequencing of South Korean male patients with stage IV CRC. Also, functional assays should be conducted to determine the efficiency of the Wnt pathway in the presence of *WIF1*, *SFRP4*, *UCA1*, and *CRNDE*. Although at the RNA level, these players are highly expressed; there may be post-transcriptional and post-translational modifications that are not shown by this analysis. Very little is known about the mechanism of action of lncRNAs *UCA1* and *CRNDE*. Further investigation should delve into the functions of these lncRNAs to understand their precise role in the Wnt pathway. When the roles for *UCA1* and *CRNDE* are elucidated, they may be potential therapeutic targets. Inhibiting *UCA1* and *CRNDE* may allow *WIF1* to effectively inhibit the Wnt pathways, which would decrease the cell proliferation, cell polarity, and migration.

Finally, further research is needed on the mechanisms of the remaining differentially expressed inhibitors found in this dataset: *SFRP4*, *DKK2*, *DKK4*, *APCDD1*, and *NOTUM*. The full elucidation of the mechanisms and pathways of the inhibitors in the Wnt pathway would be a powerful tool for new diagnostics and therapeutics advances in the case of stage IV CRC.

## Supplementary Material

Supplemental data

Supplemental data

Supplemental data

Supplemental data

Supplemental data

Supplemental data

## Abbreviations Used

APC: adenomatous polyposis coli
CRC: colorectal cancer
CRNDE: colorectal neoplasia differentially expressed
DEG: differentially expressed gene
FAP: familial adenomatous polyposis
Fzd: Frizzled
GEO: Gene Expression Omnibus
H3K27: histone H3 on lysine 27
HOTAIR: HOX transcript antisense RNA
IGV: Integrative Genomics Viewer
lncRNA: long noncoding RNA
LSD1: lysine-specific demethylase 1
NFκB: nuclear factor kappa B
PRC2: polycomb repressive complex 2
SFRP4: secreted frizzled-related protein 4
SNP: single-nucleotide polymorphism
TFBS: transcription factor binding site
UCA1: urothelial carcinoma associated 1
WIF1: Wnt inhibitory factor 1
