# Supplementary material for: Toward Colorectal Cancer Biomarkers: The Role of Genetic Variation, Wnt Pathway, and Long Noncoding RNAs
Source: OMICS. 2021 May 7;25(5):302–12. doi: 10.1089/omi.2020.0231 (PMC8110006; doi:10.1089/omi.2020.0231)
Supplement: Supplemental data [file Supp_Fig2.pdf]

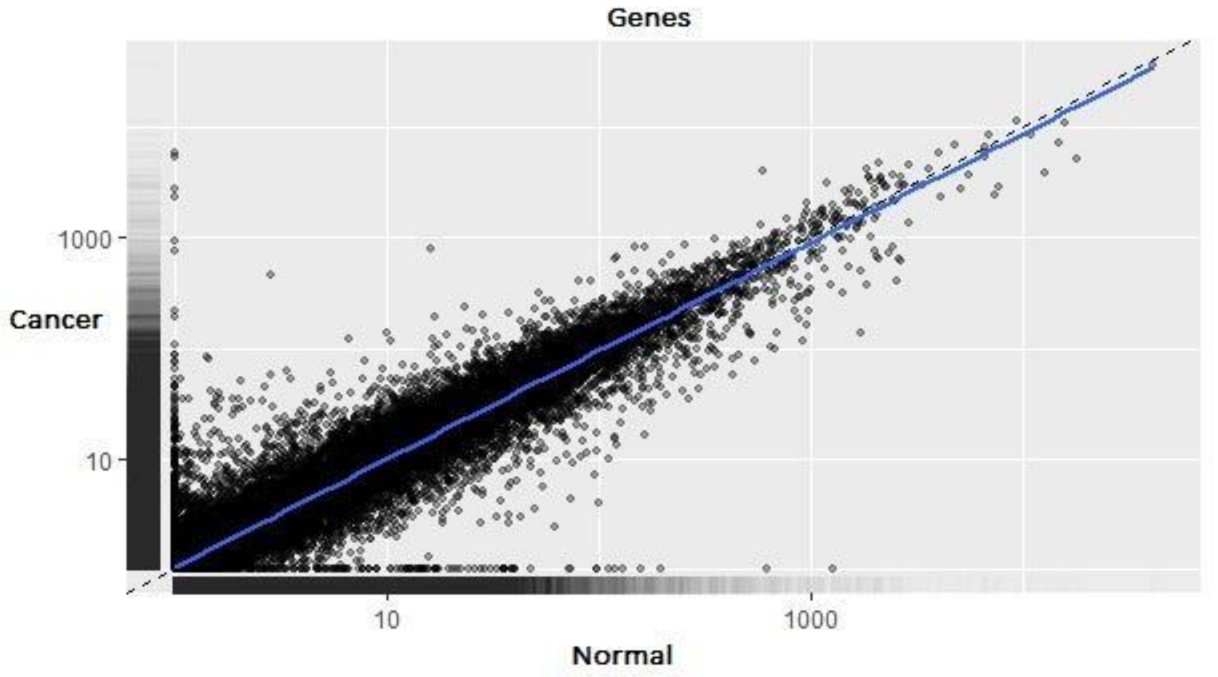

Supplementary Figure 2: Scatter Plot of Genes Expressed in Normal Colon Mucosa vs Genes Expressed in CRC by cummeRbund. The scatter plot maps the genes expressed in both conditions and in this scatter plot there is a roughly linear relationship between most of the genes expressed in both conditions. The data points that are outside of the linear portion are the genes that are differentially expressed.
