# Supplementary material for: Toward Colorectal Cancer Biomarkers: The Role of Genetic Variation, Wnt Pathway, and Long Noncoding RNAs
Source: OMICS. 2021 May 7;25(5):302–12. doi: 10.1089/omi.2020.0231 (PMC8110006; doi:10.1089/omi.2020.0231)
Supplement: Supplemental data [file Supp_Fig3.pdf]

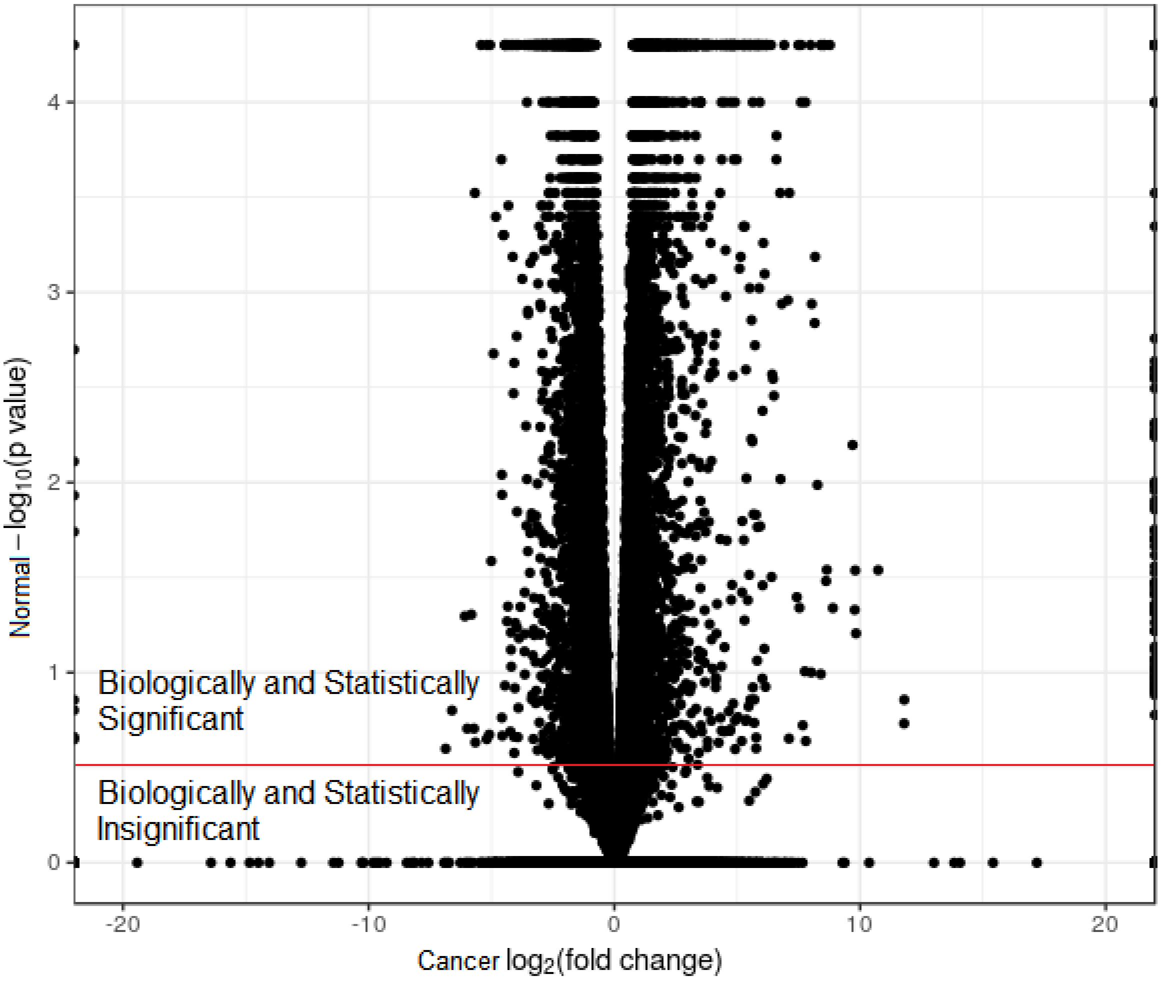

Supplementary Figure 3: Volcano Plot showing the Relationship between the Normal and Cancer Samples by cummeRbund. Using the p-value cut off of less than or equal to 0.05 and fold change cut off of greater than or equal to 0.5 and less than or equal to -0.5, the biological and statistical significance were determined. The data points above the red line show the biological and statistical significant genes.
