# Supplementary material for: Toward Colorectal Cancer Biomarkers: The Role of Genetic Variation, Wnt Pathway, and Long Noncoding RNAs
Source: OMICS. 2021 May 7;25(5):302–12. doi: 10.1089/omi.2020.0231 (PMC8110006; doi:10.1089/omi.2020.0231)
Supplement: Supplemental data [file Supp_Fig4.pdf]

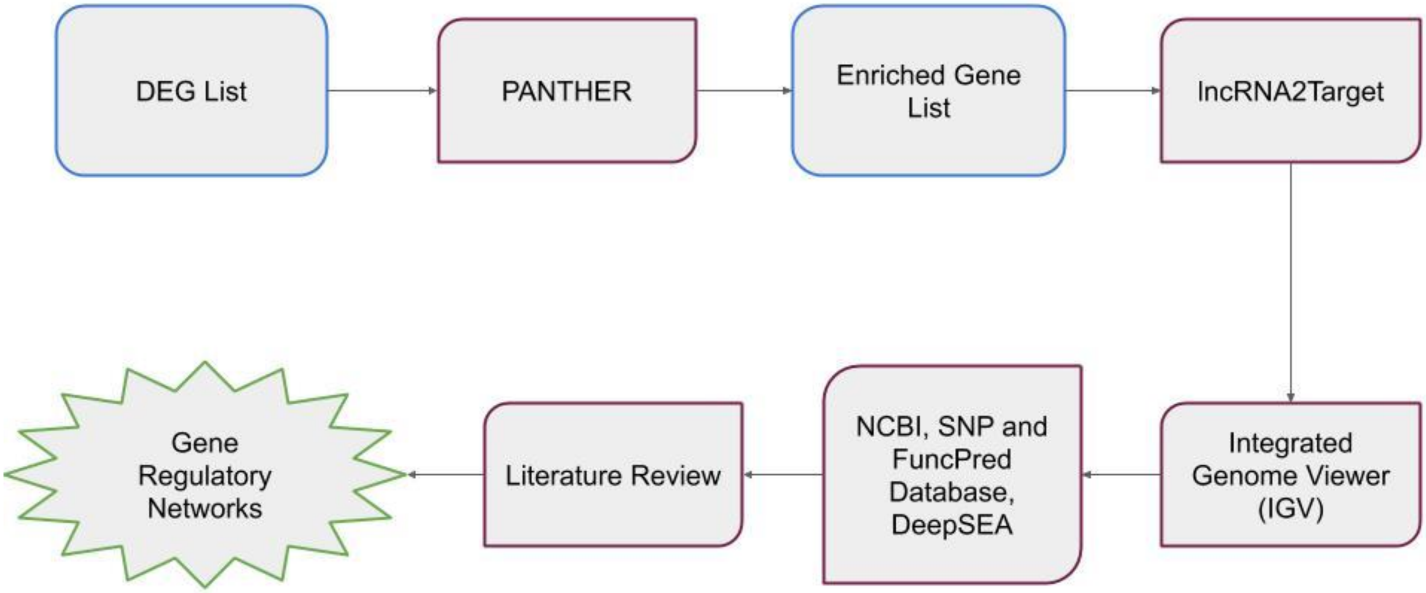

Supplementary Figure 4: DEG Analysis Pipeline. After obtaining the DEGs from the Tuxedo Suite Pipeline, the DEGs were entered into PANTHER for pathway enrichment. The associated lncRNAs of the genes of the most enriched pathways were found by using lncRNA2Target database. After determining a set of genes of interest, any genetic variants were investigated through the Integrated Genome Viewer. The SNPs found were cross referenced with NCBI, SNP database, FuncPred database, and any novel SNPs were further analyzed with DeepSEA. With this information and a thorough literature review, connections were deduced.
