# Supplementary material for: Toward Colorectal Cancer Biomarkers: The Role of Genetic Variation, Wnt Pathway, and Long Noncoding RNAs
Source: OMICS. 2021 May 7;25(5):302–12. doi: 10.1089/omi.2020.0231 (PMC8110006; doi:10.1089/omi.2020.0231)
Supplement: Supplemental data [file Supp_Fig1.pdf]

Supplementary Figures

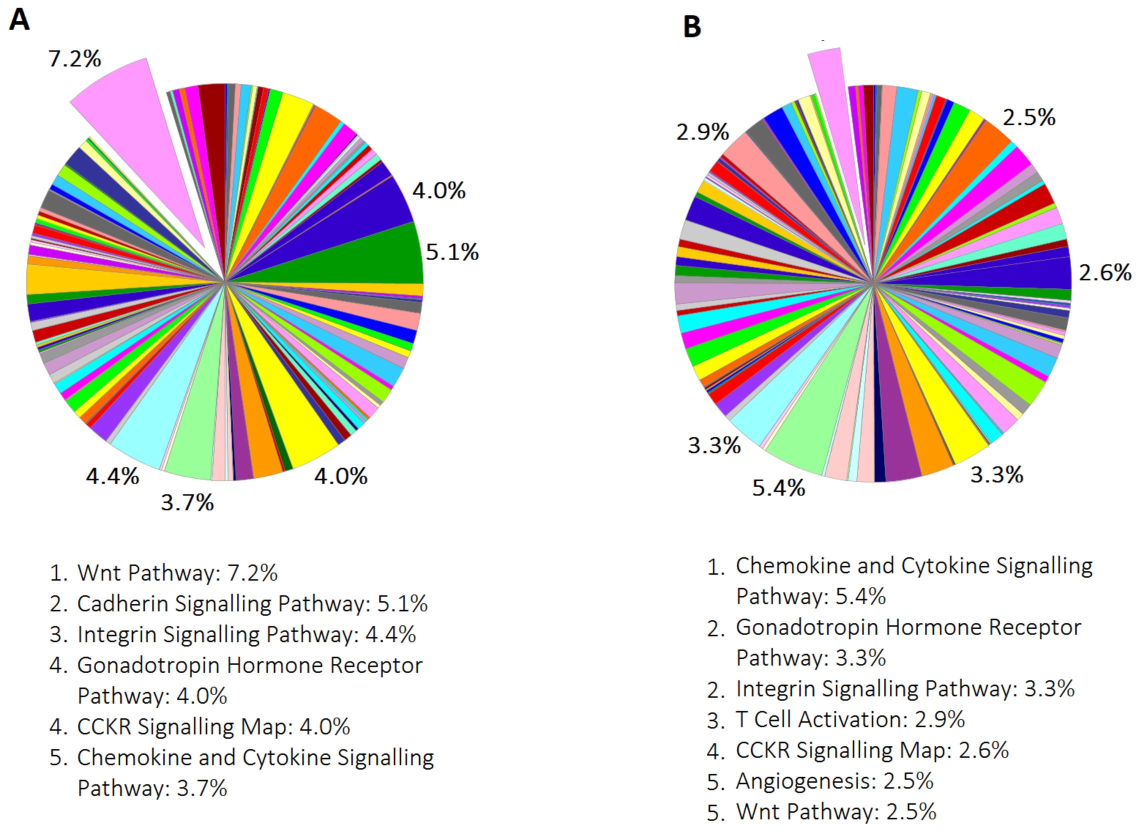

Supplementary Figure 1: Pathway enrichment analysis by PANTHER (Protein ANalysis THrough Evolutionary Relationships) database. A) This pie chart represents the pathway enrichment for only the upregulated genes. The top 5 enriched pathways are listed with their corresponding percentages. The Wnt Pathway is at the top and represents 7.2% of the genes. B) This pie chart represents the pathway enrichment for the downregulated genes. The top 5 enriched pathways are listed with their corresponding percentages. The Wnt Pathway ranks at the fifth most enriched pathway with its genes representing 2.5% of the downregulated differentially expressed genes.
