# Supplementary material for: Toward Colorectal Cancer Biomarkers: The Role of Genetic Variation, Wnt Pathway, and Long Noncoding RNAs
Source: OMICS. 2021 May 7;25(5):302–12. doi: 10.1089/omi.2020.0231 (PMC8110006; doi:10.1089/omi.2020.0231)
Supplement: Supplemental data [file Supp_Fig5.pdf]

Supplementary Figure 5: List of genes with associated log fold change and p-values.

Gene	Gene Type	Fold Change	p-value
WNT9A	Activator	-1.503	0.00075
WNT3	Activator	1.503	5.00E-05
WNT11	Activator	1.996	5.00E-05
WNT5A	Activator	2.261	5.00E-05
WNT2	Activator	5.772	0.0019
SFRP4	Inhibitor	2.211	0.00185
APCDD1	Inhibitor	2.384	5.00E-05
DKK2	Inhibitor	4.342	5.00E-05
WIF1	Inhibitor	5.165	5.00E-05
NOTUM	Inhibitor	6.812	0.00115
HOTAIR	lncRNA	5.198	0.03785
CRNDE	lncRNA	3.227	0.0032
UCA1	lncRNA	5.542	5.00E-05
